# Concurrent analysis of genome and transcriptome in one single cell

**DOI:** 10.21203/rs.3.rs-3186428/v1

**Published:** 2023-08-03

**Authors:** Johanna Heid, Ronald Cutler, Moonsook Lee, Jan Vijg, Alexander Y. Maslov

**Affiliations:** Albert Einstein College of Medicine; Albert Einstein College of Medicine; Albert Einstein College of Medicine; Albert Einstein College of Medicine; Albert Einstein College of Medicine

## Abstract

Thus far, multiple techniques for single cell analysis have been developed, yet we lack a relatively simple tool to assess DNA and RNA from the same cell at whole-transcriptome and whole-genome depths. Here we present an updated method for physical separation of cytoplasmic RNA from the nuclei, which allows for simultaneous studies of DNA and RNA from the same single cell. The method consists of three steps – 1) immobilization of a single cell on solid substrate, 2) hypotonic lysis of immobilized single cell, and 3) separation of cytosol containing aqueous phase and immobilized nucleus. We found that DNA and RNA extracted from single cell using our approach is suitable for downstream sequencing-based applications. We demonstrated that the coverage of transcriptome and genome sequencing data obtained after DNA/RNA separation is similar to that observed without separation. We also showed that the separation procedure does not create any noticeable bias in observed mutational load or mutation spectra. Thus, our method can serve as a tool for simultaneous complex analysis of the genome and transcriptome, providing necessary information on the relationship between somatic mutations and the regulation of gene expression.

## Introduction

The advent and rapid development of single cell sequencing technologies permitting fast and accurate analysis of individual whole genomes resulted in an avalanche of experimental evidence on the pivotal role of somatic mutations in human aging and diseases such as cancer ^1,2^. Arguably, single cell-based approaches played the most important role in our progress in study of somatic mutation as they allow us to accurately assess random somatic mutations ^3,4^. These studies clearly demonstrated that a healthy somatic cell contains several hundred unique mutations, a number which increases due to multiple factors over the course of life and reaches thousands towards the end of life ^5,6^. However, despite evidence of increasing mutations during aging, it still remains unclear whether these changes in genome are translated to changes in cellular function. It has become apparent now that complete understanding of these mechanisms is only possible by comprehensive analysis of changes in both genome and transcriptome of a single cell, the only methodology that allows the establishment of direct links between mutations and their functional implementation.

There are several approaches available for separation and further analysis of DNA and RNA contents of one single cell. Many of these methods are based on capture of mRNA molecules using oligo-dT probes followed by immobilization and separation of captured transcripts from genomic DNA ^7–9^. An alternative approach was implemented by simultaneous isolation of genomic DNA and total RNA (SIDR) where RNA-containing cytoplasm is released into aqueous phase by hypotonic lysis and then separated from the immobilized nucleus by simple aspiration ^10^. However, practical application of SIDR is limited by availability of cell-surface specific antibodies conjugated to magnetic beads. Here we introduce a simple modification to SIDR allowing isolation and parallel analysis of DNA and RNA from a single cell using the CellRaft AIR system by Cell Microsystems as a mean to immobilize individual cells prior to lysis. We demonstrate that DNA and RNA separated with the modified approach are suitable for downstream applications and that this procedure does not introduce any significant bias to the results of DNA and RNA analysis.

## Results

### Single cell isolation and fractionation

**Figure 1:**
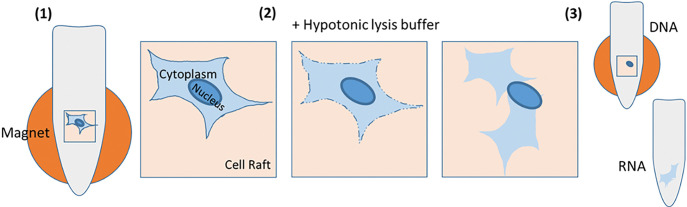
Separation of DNA and RNA utilizing the CellRaft system. A raft with a single cell is selected and transferred to a PCR tube. The tube is placed on a magnetic stand to hold the raft into place (1). Hypotonic lysis buffer is added (2) leading to disruption of the cell membrane and release of the cytoplasm into the buffer. The supernatant is transferred to a new tube while the nucleus with the DNA remains on the raft (3). Both tubes can be stored at −80C until further processing.

Isolation of primary human IMR90 fibroblasts was conducted using the automated Air Raft system by Cell Microsystems. This machine allows direct visualization and identification of cells plated and adhered to the CytoSort Array, composed of thousands of microwells (so-called CellRafts). The CellRafts are magnetic and can be individually selected and released from the array. When an individual cell is identified on the surface of a CellRaft, it is then picked and transferred to a PCR tube using a magnetic wand. Once deposited into tube, the individual cell is then exposed to the hypotonic lysis buffer (HLB) to disrupt the cell membrane and release cytoplasm into aqueous phase as described previously by others 10. The DNA-containing nucleus remains embedded into cytoskeleton and attached onto the CellRaft. Next, the tube is placed on a magnetic stand to immobilize the CellRaft. The RNA-containing supernatant is then aspirated and transferred to a separate tube, leaving the DNA-containing nucleus in the original tube (Fig. 1). With this, DNA and RNA are physically separated from each other and can be further processed either immediately or stored at −80C until needed.

### DNA/RNA separation does not affect single cell RNA-seq results

**Figure 2:**
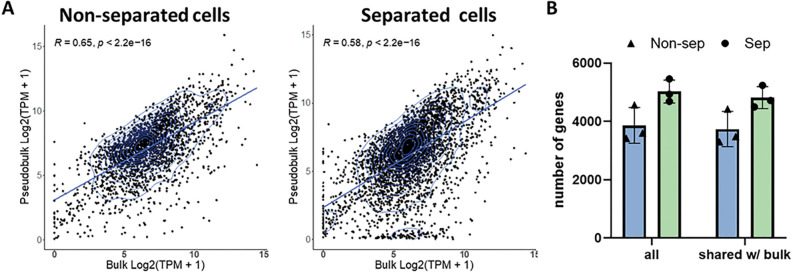
Single cell mRNA expression correlates with mRNA expression of bulk cells. A) Correlation of non-separated cells and separated cells based on genes of single cells. B) Total number of genes as well as genes overlapping with genes of bulk detected from non-separated (blue) and separated (green) cells.

As a first step, we tested if DNA/RNA separation procedure introduce a bias in results of gene expression analysis. We were able to successfully convert single cell cytoplasmic RNA into cDNA after DNA/RNA separation via hypotonic lysis and generate sequencing libraries (Supp. Figure 1). We also constructed RNA-seq libraries from intact single cells, without DNA/RNA separation, as a control. In total we analyzed 3 native cells without separation and 3 cells after DNA/RNA separation procedure. First, we tested if separation procedure affects relative representation of different transcripts in RNA-seq data. We reasoned that single-cell transcriptomes combined in one pseudo-bulk should provide a set of data similar to the bulk RNA-seq of thousands of cells and, hence, may serve as a measure for accuracy of transcriptome assessment. We composed two pseudo-bulk data sets, one containing single cell RNA-seq data after DNA/RNA separation and the other without that. Only genes with at least 3 counts in each analyzed cell were included in pseudo-bulks. We found that these two pseudo-bulks have similar levels of correlation with RNA-seq data (R = 0.65 and R = 0.58, non-separated and separated, respectively; Fig. 2A). Moreover, we compared the correlation of the individual cells with the bulk and found slightly higher correlation in separated cells (**Supp.** Figure 2). Finally, we assessed the total number of genes detected in single cells as well as the number of genes shared between single cells and the bulk and did not see a significant difference between separated and naïve cells (Fig. 2B). Thus, we found that DNA/RNA separation procedure does not introduce any significant bias in single-cell transcriptome analysis results and transcriptomics data obtained after separation procedure can be confidently used.

### DNA/RNA separation does not affect single cell genome analysis

We first tested if DNA from immobilized nuclei can be successfully amplified. For this purpose, after separation of RNA-containing aqueous fraction remaining nuclei attached to CellRafts were subjected to whole genome amplification (WGA) using our scMDA approach ^4^. We found that the DNA yield of scMDA procedure was lower between cells with and without DNA/RNA separation (248ng/μl ± 2 ng/μl vs 442ng/μl ± 36ng/μl, respectively). We performed locus drop-out test (LDO), a qPCR-based approach measuring relative representation of 8 different targets distributed across the genome ^3^, to assess the uniformity of the whole genome amplification.. The LDO test also did not reveal any significant differences in quality of WGA products with and without DNA/RNA separation (Table 1).

We also did not observe a statistically significant difference in the rates of genome coverage in single cell genome sequencing data obtained from cells with and without DNA/RNA separation procedure (**Supp.** Figure 3).

**Figure 3:**
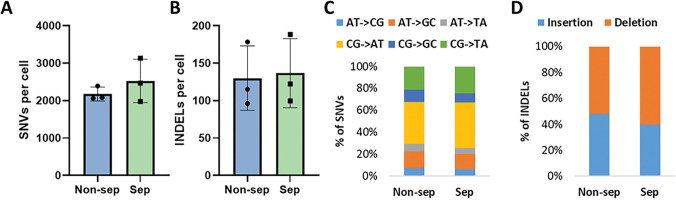
The DNA / RNA separation process does neither influence frequency of mutations nor mutational spectra. A) Number of SNVs and B) Number of INDELs detected in non-separated (blue) and separated single cells (green). C) The distribution of SNVs does not vary significantly between separated and non-separated cells. D) The spectrum of INDELs is not significantly impacted by the DNA/RNA separation procedure. N=3

The frequency of somatic SNVs in single cells subjected to DNA/RNA separation was 2528 ± 518 SNVs per cell, which is not significantly different from that observed in intact single cells (2177 ± 185 SNVs per cell) (Fig. 3A). Also, no difference between non-separated and separated cells was observed in the levels of INDELs: non-separated cells contained 130 ± 43 INDELs and separated 136 ± 46 INDELs per cell (Fig. 3B).

To further investigate a possible separation effect in more detail, we assessed the mutational spectra of sepaterated and non-separated cells. The distribution of SNVs did not change significantly in separated cells compared to non-separated cells (Fig. 3C). Moreover, the ratio of insertions and deletions was not affected by the separation procedure (Fig. 3D). Hence, we concluded that the separation method is not mutagenic and does not introduce any significan bias to the results of analysis of mutational burden and spectra.

## Discussion

Here we present a simplified method for DNA/RNA separation that allows for simultaneous investigation of gene expression profiles and mutation patterns in the same single cell. When comparing bulk RNA-seq data and pseudo-bulks compiled from single-cell RNA-seq results of cells with and without DNA/RNA separation, we found similar levels of correlation. In addition, the DNA/RNA separation procedure did not affect the number of identified genes in the single-cell RNA-seq or the proportion of identified genes overlapping with the bulk RNA-seq.

Analysis of single cell WGS results revealed similar levels of observed somatic mutations, both SNV and small INDELs, in genomic DNA after separation from RNA and without separation. Spectra of identified somatic SNVs as well as distribution of insertions and deletions also remained similar between these two cohorts. While we observed more variance in genomic coverage in the separated cells than in the non-separated ones, the resulting WGS data was well qualified for further analysis and accuracy of variant calling was not affected.

Thus, we described a simplified method for single cell DNA/RNA separation allowing their concurrent analysis and demonstrated that the separation procedure per se does not influence observed gene expression patterns, mutation frequency and spectra. Of note, the manual version of the CellRaft method is also available that makes this approach applicable in virtually any laboratory. These open up new possibilities to study the direct relation between mutations and their effect on gene expression in a wide range of cells. Moreover, we also successfully used the DNA fraction as input for bisulfite treatment for the assessment of DNA methylation pattern after a previously published protocol for single cells ^11^ allowing to directly investigate the regulation of gene expression in a particular cell in a comprehensive manner. Taken together we present a powerful tool for studying single cells for a refined insight on processes in any given cell.

## Materials and Methods

### Cell culture

Commercially available IMR90 human lung fibroblasts (ATCC) were cultured in EMEM (ATCC) supplemented with 10% FBS (Gibco) and 1% Pen/Strep (Gibco) at 37°C, 3% O2 and 10% CO2. Cell viability, apoptosis rate and cell number were assessed with the Guava EasyCyte cytometer. The CellRaft array was prepared following the manufacturer’s instruction and washed three times for 3 minutes with warm PBS before addition of cell suspension. 1000 cells were seeded in 1ml for one CellRaft array. Cells were allowed to settle for 3–4 hours at 37°C before isolation. Before isolation the raft was carefully washed with warm medium to remove potential debris and dead cells.

### DNA/RNA separation

Single cells were isolated using the CellRaft AIR system (Cell Microsystems) and individual rafts containing one cell were deposited in PCR tubes with 2.5ul PBS. After isolation, tubes were quickly checked under a microscope to ensure proper deposition of the raft into the tube. Then tubes were put on wet-ice and 9 μl freshly prepared hypotonic lysis buffer (HLB) was added. The HLB composition was 0.2% Triton X-100 (Sigma-Aldrich) and 0.5% RNAse Inhibitor (Takara) in nuclease-free water ^10^. Tubes containing cells and HLB were briefly spun and incubated for 10 minutes at room temperature. 2 minutes before incubation time was over, tubes were briefly spun again and placed in magnetic stand. The supernatant was transferred to a new tube and placed immediately on dry ice. 2.5 μl PBS were added to the remaining cell raft and then also placed on dry ice. Tubes with DNA and RNA respectively were stored at −80°C until further processing.

### Library preparation from single cells

For RNA sequencing, cDNA was prepared using the Smart seq 4 kit (Takara) using manufacturer recommended protocol. Briefly, the RNA of the cytosolic fraction(11μl) was used as input for conversion. The product was quantified via Qubit (ThermoFisher Scientific) and quality was assessed using a HS5000 screen tape on the TapeStation (Agilent). Following this, sequencing libraries were prepared utilizing the Nextera XT kit (Illumina) with a 4minute tagmentation step and 13 cycles of amplification followed by a purification using AMPure beads (Beckman Coulter). Libraries for whole genome sequencing (WGS) were prepared according to the previously described scMDA protocol using a NEBNext Ultra II FS kit (NEB) ^4^. For quality control purposes, a locus drop out test (LDO) was performed using primers and SYBR reagent ^12^. Library size was assessed with a TapeStation (Agilent) using HS5000 screen tape for quality control of cDNA and HS1000 screen tape for all final libraries. All RNA-Seq and WGS libraries were sequenced on an Illumina HiSeq platform by Novogene Corp Inc., CA.

### Computational analysis

For RNA-Seq processing, paired-end 150 bp raw reads were first trimmed using Trim Galore ^13^ and quality control performed using FastQC ^14^, STAR ^15^ was used for alignment, duplicates were removed using Picard (http://broadinstitute.github.io/picard/) and RSEM ^16^ was used for quantification. Gene level counts and effective lengths were extracted using tximport ^17^ into the R environment. Genes with evidence of consistent expression, those with at least 3 counts in all bulk samples or single cells of the same condition, were used for downstream analysis. Pseudo bulk samples were created by summing counts across cells of the same condition. Transcripts per million (TPM) were calculated for bulk and pseudo bulk samples after normalization using effective gene lengths.

For WGS processing, paired-end reads were trimmed using Trim Galore and quality control performed using FastQC. Alignment was performed with BWA MEM ^18^ and variants were called using SCcaller ^4^.

## Figures and Tables

**Table 1:** LDO results of separated and non-separated single cells. ∆∆CT values greater than 0.2 mark the passing of the particular genomic loci.

	sep_1	sep_2	sep_3	non-sep1	non-sep2	non-sep3
target 1	1.562419	1.16816	0.757456	0.20583	0.462665	0.516902
target 2	0.378329	0.924101	0.385863	0.309555	0.255154	0.138418
target 3	1.040357	0.244198	0.002706	0.463318	0.129319	0.959483
target 4	0.073058	2.03288	0.715105	0.515424	0.310383	0.23392
target 5	1.015064	0.16847	0.328062	0.629856	3.384569	0.695215
target 6	0.69544	4.320695	0.187688	0.512731	1.674983	0.411174
target 7	1.462495	0.36903	0.308607	0.828195	0.915885	0.453706
target 8	1.792035	0.791462	2.27111	0.239909	0.466191	0.295373

## Data Availability

We uploaded our data sets to publicly available servers and are currently awaiting accession numbers. Raw reads will be available here: https://www.ncbi.nlm.nih.gov/sra under PRJNA975175
